# The Illinois State Medical Society—Committee on Diseases of the Eye

**Published:** 1865-01

**Authors:** 


### THE ILLINOIS STATE MEDICAL SOCIETY.

### COMMITTEE ON DISEASES OF THE EYE.

The Committee would most respectfully request members of the Society and of the profession generally, to contribute the report of important cases, and papers upon any subject in Ophthalmology. The chairman of the Committee would respectfully solicit the record of all facts relating to epidemics of Conjunctivitis in different parts of the State—the condition of the atmosphere and soil, the habits and occupations of the patients, and the severity and number of cases. He would also solicit the history of cases of injury of one eye followed by sympathetic ophthalmitis in the other eye.

E. L. Holmes, Chicago. R. E. McVey, Waverly.
J. S. Whitmire, Metamora. F. B. Haller, Vandalia.

